# National, Regional, State, and Selected Local Area Vaccination Coverage Among Adolescents Aged 13–17 Years — United States, 2019

**DOI:** 10.15585/mmwr.mm6933a1

**Published:** 2020-08-21

**Authors:** Laurie D. Elam-Evans, David Yankey, James A. Singleton, Natalie Sterrett, Lauri E. Markowitz, Charnetta L. Williams, Benjamin Fredua, Lucy McNamara, Shannon Stokley

**Affiliations:** ^1^Immunization Services Division, National Center for Immunization and Respiratory Diseases, CDC; ^2^Oak Ridge Institute for Science and Education, Oak Ridge, Tennessee; ^3^Division of Viral Diseases, National Center for Immunization and Respiratory Diseases, CDC; ^4^Leidos Health, Inc., Atlanta, Georgia; ^5^Division of Bacterial Diseases, National Center for Immunization and Respiratory Diseases, CDC.

Three vaccines are recommended by the Advisory Committee on Immunization Practices (ACIP) for routine vaccination of adolescents aged 11–12 years to protect against 1) pertussis; 2) meningococcal disease caused by types A, C, W, and Y; and 3) human papillomavirus (HPV)-associated cancers (*1*). At age 16 years, a booster dose of quadrivalent meningococcal conjugate vaccine (MenACWY) is recommended. Persons aged 16–23 years can receive serogroup B meningococcal vaccine (MenB), if determined to be appropriate through shared clinical decision-making. CDC analyzed data from the 2019 National Immunization Survey-Teen (NIS-Teen) to estimate vaccination coverage among adolescents aged 13–17 years in the United States.* Coverage with ≥1 dose of HPV vaccine increased from 68.1% in 2018 to 71.5% in 2019, and the percentage of adolescents who were up to date^†^ with the HPV vaccination series (HPV UTD) increased from 51.1% in 2018 to 54.2% in 2019. Both HPV vaccination coverage measures improved among females and males. An increase in adolescent coverage with ≥1 dose of MenACWY (from 86.6% in 2018 to 88.9% in 2019) also was observed. Among adolescents aged 17 years, 53.7% received the booster dose of MenACWY in 2019, not statistically different from 50.8% in 2018; 21.8% received ≥1 dose of MenB, a 4.6 percentage point increase from 17.2% in 2018. Among adolescents living at or above the poverty level,^§^ those living outside a metropolitan statistical area (MSA)^¶^ had lower coverage with ≥1 dose of MenACWY and with ≥1 HPV vaccine dose, and a lower percentage were HPV UTD, compared with those living in MSA principal cities. In early 2020, the coronavirus disease 2019 (COVID-19) pandemic changed the way health care providers operate and provide routine and essential services. An examination of Vaccines for Children (VFC) provider ordering data showed that vaccine orders for HPV vaccine; tetanus toxoid, reduced diphtheria toxoid, and acellular pertussis vaccine (Tdap); and MenACWY decreased in mid-March when COVID-19 was declared a national emergency (Supplementary Figure 1, https://stacks.cdc.gov/view/cdc/91795). Ensuring that routine immunization services for adolescents are maintained or reinitiated is essential to continuing progress in protecting persons and communities from vaccine-preventable diseases and outbreaks.

NIS-Teen is a random-digit-dial telephone survey** conducted annually to monitor vaccination coverage among adolescents aged 13–17 years in the 50 states, the District of Columbia, selected local areas, and selected U.S. territories.^††^ Sociodemographic information is collected during the telephone interview with a parent or guardian, and a request is made for consent to contact the adolescent’s vaccination provider(s). If consent is obtained, a questionnaire is mailed to the vaccination provider(s) to request the adolescent’s vaccination history. Vaccination coverage estimates are determined from these provider-reported immunization records. This report provides vaccination coverage estimates on 18,788 adolescents aged 13–17 years.^§§^ The overall Council of American Survey Research Organizations (CASRO)^¶¶^ response rate was 19.7%, and 44.0% of adolescents for whom household interviews were completed had adequate provider data.

Data were weighted and analyzed to account for the complex sampling design.*** T-tests were used to assess vaccination coverage differences between sociodemographic subgroups. P-values <0.05 were considered statistically significant. All analyses were conducted using SAS-callable SUDAAN (version 11; RTI International).

## National Vaccination Coverage

In 2019, 71.5% of adolescents aged 13–17 years had received ≥1 dose of HPV vaccine, and 54.2% had completed the HPV vaccination series and were considered HPV UTD (Table 1, Figure). Increases from 2018 in ≥1 dose HPV vaccine coverage and HPV UTD status were observed for females and for males. Coverage with ≥1 dose of MenACWY increased by 2.3 percentage points to 88.9%. Coverage with ≥2 MenACWY doses among adolescents aged 17 years was 53.7%, similar to that in 2018 (50.8%). Coverage with ≥1 dose of MenB among adolescents aged 17 years increased from 17.2% in 2018 to 21.8% in 2019. Coverage with ≥1 dose of Tdap remained stable and high (90.2%). Coverage exceeded 90% for ≥2 doses measles, mumps, and rubella vaccine (MMR), ≥3 doses of hepatitis B vaccine, and ≥1 and ≥2 doses of varicella vaccine among adolescents without a history of varicella disease.^†††^

**TABLE 1:** Estimated vaccination coverage with selected vaccines and doses among adolescents aged 13–17* years, by age at interview — National Immunization Survey–Teen (NIS-Teen), United States, 2019

Vaccine	Age at interview, yrs					Total	
	% (95% CI)^†^						
	13	14	15	16	17	2019	2018
	(n = 3,927)	(n = 4,007)	(n = 3,753)	(n = 3,753)	(n = 3,348)	(n = 18,788)	(n = 18,700)
**Tdap^§^ ≥1 dose**	89.0 (87.2–90.6)	91.8 (89.6–93.5)^¶^	91.4 (89.6–92.9)	89.5 (87.4–91.3)	88.9 (85.3–91.7)	90.2 (89.2–91.1)	88.9 (88.0–89.7)
MenACWY**							
≥1 dose	87.7 (86.0–89.3)	91.2 (89.6–92.5)^¶^	88.3 (86.2–90.1)	88.3 (85.8–90.4)	88.9 (85.9–91.4)	88.9 (88.0–89.8)^††^	86.6 (85.6–87.5)
≥2 doses**^§§^**	NA	NA	NA	NA	53.7 (49.9–57.4)	53.7 (49.9–57.4)	50.8 (47.7–53.8)
HPV^¶¶^ vaccine							
All adolescents							
≥1 dose	66.9 (64.1–69.6)	73.6 (70.8–76.3)^¶^	72.1 (69.1–75.0)^¶^	71.2 (68.1–74.0)^¶^	73.1 (69.7–76.3)^¶^	71.5 (70.1–72.8)^††^	68.1 (66.8–69.3)
HPV UTD***	45.3 (42.1–48.5)	52.2 (48.6–55.8)^¶^	58.6 (55.3–61.8)^¶^	57.6 (54.4–60.8)^¶^	57.1 (53.2–60.8)^¶^	54.2 (52.7–55.8)^††^	51.1 (49.8–52.5)
Females							
≥1 dose	68.4 (64.0–72.5)	75.1 (71.4–78.5)^¶^	75.6 (71.6–79.2)^¶^	71.9 (67.1–76.3)	74.9 (70.0–79.2)^¶^	73.2 (71.3–75.0)^††^	69.9 (68.1–71.6)
HPV UTD	48.9 (43.9–53.9)	53.0 (48.0–57.9)	61.6 (57.0–66.0)^¶^	61.5 (56.8–66.0)^¶^	59.2 (53.6–64.5)^¶^	56.8 (54.6–59.0)^††^	53.7 (51.8–55.6)
Males							
≥1 dose	65.4 (61.8–68.8)	72.2 (67.8–76.1)^¶^	68.9 (64.3–73.1)	70.4 (66.4–74.1)	71.6 (66.6–76.1)^¶^	69.8 (67.9–71.7)^††^	66.3 (64.6–68.0)
HPV UTD	41.5 (37.9–45.3)	51.5 (46.2–56.7)^¶^	55.7 (51.1–60.2)^¶^	53.9 (49.5–58.2)^¶^	55.2 (49.9–60.4)^¶^	51.8 (49.7–53.9)^††^	48.7 (46.8–50.6)
**MenB ≥1 dose^†††^**	NA	NA	NA	NA	21.8 (18.9–24.9)	21.8 (18.9–24.9)^††^	17.2 (14.9–19.9)
**MMR ≥2 doses**	93.0 (91.1–94.4)	91.2 (88.1–93.5)	93.3 (91.7–94.6)	91.2 (89.0–92.9)	90.7 (87.5–93.2)	91.9 (90.8–92.8)	91.9 (91.2–92.6)
**Hepatitis A vaccine ≥2 doses^§§§^**	84.1 (81.6–86.2)	79.8 (76.7–82.6)^¶^	78.1 (75.3–80.6)^¶^	71.8 (68.9–74.5)^¶^	71.9 (68.1–75.4)^¶^	77.1 (75.8–78.4)^††^	73.6 (72.4–74.7)
**Hepatitis B vaccine ≥3 doses**	92.1 (90.1–93.7)	91.6 (88.6–93.8)	92.8 (91.1–94.2)	90.7 (88.5–92.5)	90.8 (87.4–93.4)	91.6 (90.6–92.6)	92.1 (91.3–92.8)
Varicella							
History of varicella^¶¶¶^	6.8 (5.4–8.5)	8.4 (7.0–10.0)	9.6 (8.0–11.4)^¶^	10.4 (8.7–12.3)^¶^	10.4 (8.8–12.4)^¶^	9.1 (8.4–9.9)^††^	11.9 (11.0–12.7)
No history of varicella disease							
≥1 dose vaccine	96.0 (94.9–96.8)	94.7 (92.1–96.5)	95.8 (94.6–96.7)	94.4 (92.3–95.9)	95.0 (92.2–96.9)	95.2 (94.3–95.9)	94.9 (94.3–95.4)
≥2 doses vaccine	91.6 (89.6–93.2)	91.0 (87.7–93.5)	92.5 (90.9–93.8)	90.1 (87.7–92.0)	87.8 (84.1–90.7)	90.6 (89.5–91.7)	89.6 (88.7–90.4)
**Varicella disease or received ≥2 varicella vaccine doses**	92.2 (90.3–93.7)	91.8 (88.7–94.0)	93.2 (91.8–94.4)	91.1 (88.9–92.9)	89.1 (85.7–91.7)	91.5 (90.4–92.4)	90.8 (90.0–91.6)

**Abbreviations:** CI = confidence interval; HPV = human papillomavirus; MenACWY = quadrivalent meningococcal conjugate vaccine; MenB = serogroup B meningococcal vaccine; MMR = measles, mumps, and rubella vaccine; NA = not applicable; Tdap = tetanus toxoid, reduced diphtheria toxoid, and acellular pertussis vaccine; UTD = up-to-date.

* Adolescents (N = 18,788) in the 2019 NIS-Teen were born during January 2001–February 2007.

**^†^** Estimates with 95% CIs >20 might not be reliable.

^§^ Includes percentages receiving Tdap at age ≥10 years.

^¶^ Statistically significant difference (p<0.05) in estimated vaccination coverage by age; reference group was adolescents aged 13 years.

** Includes percentages receiving MenACWY or meningococcal-unknown type vaccine.

^††^ Statistically significant difference (p<0.05) compared with 2018 NIS-Teen estimates.

^§§^ ≥2 doses of MenACWY or meningococcal-unknown type vaccine. Calculated only among adolescents aged 17 years at interview. Does not include adolescents who received 1 dose of MenACWY at age ≥16 years.

^¶¶^ HPV vaccine, nine-valent (9vHPV), quadrivalent (4vHPV), or bivalent (2vHPV). For ≥1 dose and HPV UTD measures, percentages are reported among females and males combined (N = 18,788) and for females only (N = 8,916) and males only (N = 9,872).

*** HPV UTD includes those with ≥3 doses, and those with 2 doses when the first HPV vaccine dose was initiated before age 15 years and there was at least 5 months minus 4 days between the first and second dose. This update to the HPV recommendation occurred in December of 2016.

^†††^ ≥1 dose of MenB, administered based on individual clinical decision; calculated only among adolescents aged 17 years at interview.

^§§§^ In July 2020, ACIP revised recommendations for hepatitis A vaccination to include catch-up vaccination for children and adolescents aged 2–18 years who have not previously received hepatitis A vaccine at any age (http://dx.doi.org/10.15585/mmwr.rr6905a1).

^¶¶¶^ By parent/guardian report or provider records.

**FIGURE:**
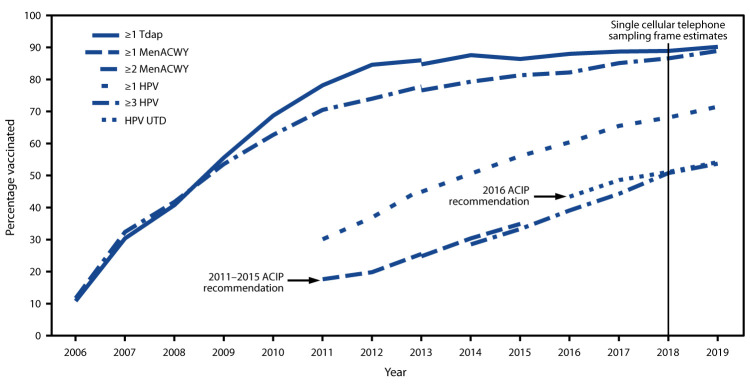
Estimated vaccination coverage with selected vaccines and doses* among adolescents aged 13–17 years, by survey year and Advisory Committee on Immunization Practices (ACIP) recommendations^†^ — National Immunization Survey-Teen (NIS-Teen)^§^^,^^¶^ — United States, 2006–2019 **Abbreviations**: HPV = human papillomavirus; MenACWY = quadrivalent meningococcal conjugate vaccine; Tdap = tetanus toxoid, reduced diphtheria toxoid, and acellular pertussis vaccine; UTD = up-to-date. *≥1 dose Tdap at or after age 10 years; ≥1 dose MenACWY or meningococcal-unknown type vaccine; ≥2 doses MenACWY or meningococcal-unknown type vaccine, calculated only among adolescents aged 17 years at time of interview. Does not include adolescents who received their first and only dose of MenACWY at or after age 16 years; HPV vaccine, nine-valent (9vHPV), quadrivalent (4vHPV) or bivalent (2vHPV). The routine ACIP recommendation for HPV vaccination was made for females in 2006 and for males in 2011. Because HPV vaccination was recommended for boys in 2011, coverage for all adolescents was not measured before that year. HPV UTD includes those with ≥3 doses, and those with 2 doses when the first HPV vaccine dose was initiated before age 15 years and at least 5 months minus 4 days elapsed between the first and second dose. † ACIP revised the recommended HPV vaccination schedule in late 2016. The recommendation changed from a 3-dose to 2-dose series with appropriate spacing between receipt of the first and second dose for immunocompetent adolescents initiating the series before the 15th birthday. Three doses are still recommended for adolescents beginning the series between ages 15 and 26 years. Because of the change in recommendation, the graph includes estimates for ≥3 doses HPV from 2011 to 2015 and the HPV UTD estimate from 2016–2019. The routine ACIP recommendation for HPV vaccination was made for females in 2006 and for males in 2011. Because HPV vaccination was not recommended for males until 2011, coverage for all adolescents was not measured before that year. ^§^ NIS-Teen revised the adequate provider definition (APD) in 2014 and retrospectively applied that definition to 2013 data. Estimates using different APD definitions might not be directly comparable. ^¶^ NIS-Teen moved from a dual landline and cellular telephone sampling frame to a single cellular telephone sampling frame in 2018.

## Vaccination Coverage by Selected Characteristics

In 2019, compared with adolescents living in MSA principal cities, coverage with ≥1 dose of HPV vaccine among those living in non-MSA areas was 9.6 percentage points lower, the percentage who were HPV UTD was 9.8 percentage points lower, and coverage with ≥1 dose of MenACWY was 5.1 percentage points lower. These disparities were only observed among adolescents living at or above the poverty level (Table 2). Coverage with all vaccine doses recommended for adolescents varied by jurisdiction, with differences ranging from 15 percentage points for ≥1 Tdap dose to 48.4 percentage points for being HPV UTD (Supplementary Table, https://stacks.cdc.gov/view/cdc/91797). Differences were observed in vaccination coverage by race and ethnicity^§§§^ and by health insurance status.^¶¶¶^

**TABLE 2:** Estimated vaccination coverage with selected vaccines and doses among adolescents aged 13–17* years, by metropolitan statistical area (MSA)^†^ and by poverty level — National Immunization Survey–Teen (NIS-Teen), United States, 2019

Vaccine	MSA			Below poverty level			At or above poverty level		
	% (95% CI)^§^								
	Non-MSA	MSA nonprincipal city	MSA principal city	Non-MSA	MSA nonprincipal city	MSA principal city	Non-MSA	MSA nonprincipal city	MSA principal city
	(n = 3,689)	(n = 7,745)	(n = 7,354)	(n = 607)	(n = 820)	(n = 1,376)	(n = 2,962)	(n = 6,676)	(n = 5,687)
**Tdap^¶^ ≥1 dose**	88.7 (86.7–90.5)	90.5 (89.0–91.8)	90.2 (88.5–91.7)	92.2 (88.7–94.6)	87.6 (80.3–92.4)	88.9 (86.0–91.3)	88.0 (85.6–90.1)	90.9 (89.5–92.1)	90.6 (88.4–92.4)
MenACWY**									
≥1 dose	83.5 (80.9–85.8)^††^	90.3 (89.1–91.4)	88.6 (86.8–90.2)	90.4 (87.0–93.0)	92.4 (88.5–95.1)	88.3 (84.6–91.1)	82.2 (79.0–85.0)^††^	89.7 (88.4–90.9)	88.8 (86.5–90.7)
≥2 doses^§§^	46.6 (39.2–54.2)	55.5 (49.9–61.0)	53.3 (46.9–59.5)	36.5 (23.5–51.8)^††^	51.5 (33.4–69.3)	59.5 (47.9–70.2)	48.8 (40.1–57.5)	57.1 (51.6–62.4)	50.8 (43.3–58.3)
HPV^¶¶^ vaccine									
All adolescents									
≥1 dose	64.2 (61.2–67.2)^††^	71.2 (69.2–73.1)	73.8 (71.5–75.9)	72.6 (66.8–77.7)	75.2 (67.9–81.3)	76.5 (71.4–81.0)	62.6 (59.0–66.1)^††^	70.3 (68.3–72.2)	72.4 (69.8–74.9)
HPV UTD***	47.3 (44.2–50.4)^††^	53.4 (51.2–55.7)^††^	57.1 (54.6–59.5)	54.6 (48.4–60.7)	58.8 (51.2–66.0)	58.8 (53.5–63.9)	45.4 (41.8–49.1)^††^	52.7 (50.3–55.0)^††^	56.5 (53.6–59.4)
Females									
≥1 dose	66.3 (61.7–70.7)^††^	72.3 (69.5–75.0)	75.9 (72.9–78.7)	76.2 (67.3–83.3)	73.0 (61.2–82.2)	78.4 (72.4–83.4)	64.0 (58.5–69.2)^††^	72.2 (69.6–74.7)	75.0 (71.3–78.4)
HPV UTD	49.0 (44.6–53.5)^††^	56.1 (53.0–59.2)	59.4 (55.7–63.1)	60.1 (51.0–68.4)	58.3 (47.3–68.4)	60.2 (53.3–66.7)	45.6 (40.6–50.7)^††^	55.3 (52.1–58.4)	58.7 (54.2–63.1)
Males									
≥1 dose	62.4 (58.2–66.4)^††^	70.2 (67.4–72.9)	71.4 (68.2–74.5)	69.4 (61.5–76.4)	77.5 (68.6–84.4)	74.8 (66.5–81.6)	61.3 (56.4–66.1)^††^	68.6 (65.6–71.5)	69.6 (65.9–73.1)
HPV UTD	45.7 (41.3–50.1)^††^	51.0 (47.7–54.3)	54.6 (51.4–57.8)	49.8 (41.4–58.3)	59.4 (48.7–69.3)	57.5 (49.6–65.1)	45.2 (40.0–50.5)^††^	50.5 (47.0–53.9)	54.1 (50.5–57.7)
**MMR ≥2 doses**	91.7 (90.0–93.1)	92.3 (91.0–93.3)	91.4 (89.3–93.2)	91.6 (87.6–94.4)	93.7 (90.3–95.9)	93.6 (91.6–95.1)	91.9 (90.0–93.5)	92.2 (90.8–93.4)	91.0 (88.0–93.2)
**Hepatitis A vaccine ≥2 doses^†††^**	67.4 (64.5–70.2)^††^	77.1 (75.1–78.9)	79.8 (77.6–81.9)	65.5 (59.0–71.4)^††^	82.2 (77.0–86.5)	81.1 (77.1–84.5)	68.1 (64.8–71.3)^††^	76.9 (74.9–78.9)	79.6 (76.9–82.1)
**Hepatitis B vaccine ≥3 doses**	92.5 (90.9–93.9)	92.0 (90.6–93.2)	90.9 (88.9–92.6)	92.8 (89.4–95.1)	92.6 (89.2–95.0)	91.0 (88.1–93.2)	92.7 (90.8–94.2)	92.9 (91.6–93.9)	91.4 (88.7–93.5)
Varicella									
History of varicella^§§§^	12.4 (10.3–15.0)^††^	8.3 (7.3– 9.3)	9.3 (8.1–10.6)	9.8 (7.1–13.4)	8.2 (5.9–11.2)^††^	12.3 (9.5–15.6)	13.0 (10.3–16.3)^††^	7.8 (6.8–8.9)	8.2 (7.0–9.6)
No history of varicella disease									
≥1 dose vaccine	95.0 (93.4–96.2)	95.6 (94.6–96.4)	94.7 (92.9–96.1)	95.7 (92.4–97.6)	95.0 (91.9–97.0)	95.2 (93.2–96.6)	95.2 (93.4–96.5)	95.6 (94.5–96.4)	94.8 (92.3–96.6)
≥2 doses vaccine	90.9 (89.1–92.4)	91.4 (90.2–92.6)	89.6 (87.1–91.6)	90.7 (86.2–93.9)	93.0 (89.6–95.4)	92.9 (90.7–94.6)	91.3 (89.2–93.0)	91.0 (89.6–92.3)	89.1 (85.8–91.6)
**Varicella disease or received ≥2 varicella vaccine doses**	92.0 (90.4–93.4)	92.1 (91.0–93.2)	90.5 (88.3–92.4)	91.6 (87.6–94.5)	93.6 (90.4–95.7)	93.7 (91.8–95.2)	92.4 (90.6–93.9)	91.7 (90.4–92.9)	90.0 (86.9–92.3)

**Abbreviations:** CI = confidence interval; HPV = human papillomavirus; MenACWY = quadrivalent meningococcal conjugate vaccine; MMR = measles, mumps, and rubella vaccine; Tdap = tetanus toxoid, reduced diphtheria toxoid, and acellular pertussis vaccine; UTD = up-to-date.

* Adolescents (N = 18,788) in the 2019 NIS-Teen were born January 2001 through February 2007.

^†^ MSA status was determined by household-reported county of residence and was grouped into three categories: MSA principal city, MSA nonprincipal city, and non-MSA. MSA and principal city were as defined by the U.S. Census Bureau (https://www.census.gov/programs-surveys/metro-micro/about.html). Non-MSA areas include urban populations not located within an MSA and completely rural areas.

^§^ Estimates with 95% CIs >20 might not be reliable.

^¶^ Includes percentages receiving Tdap at age ≥10 years.

** Includes percentages receiving MenACWY and meningococcal-unknown type vaccine.

^††^ Statistically significant difference (p<0.05) in estimated vaccination coverage by MSA; referent group was adolescents living in MSA principal city areas.

^§§^ ≥2 doses of MenACWY or meningococcal-unknown type vaccine. Calculated only among adolescents aged 17 years at interview. Does not include adolescents who received 1 dose of MenACWY at age ≥16 years.

^¶¶^ HPV vaccine, nine-valent (9vHPV), quadrivalent (4vHPV), or bivalent (2vHPV).

*** HPV UTD includes those who’ve received ≥3 doses and those with 2 doses when the first HPV vaccine dose was initiated before age 15 years and there was at least 5 months minus 4 days between the first and second dose. This update to the HPV recommendation occurred in December of 2016.

^†††^ In July 2020, ACIP revised recommendations for Hepatitis A vaccination to include catch-up vaccination for children and adolescents aged 2–18 years who have not previously received Hepatitis A vaccine at any age (http://dx.doi.org/10.15585/mmwr.rr6905a1).

^§§§^ By parent/guardian report or provider records.

## Trends in HPV Vaccination by Birth Cohort

HPV vaccination initiation by age 13 years increased an average of 5.3 percentage points for each consecutive birth year, from 19.9% among adolescents born in 1998 to 62.6% among those born in 2006 (Supplementary Figure 2, https://stacks.cdc.gov/view/cdc/91796). Being HPV UTD by age 13 years increased an average of 3.4 percentage points for each consecutive birth year, from 8.0% among adolescents born in 1998 to 35.5% among those born in 2006.

## Discussion

In 2019, coverage with HPV vaccine and with MenACWY improved compared with coverage in 2018. Improvements in ≥1 dose HPV and HPV UTD vaccination coverage were observed among females and males. In addition, more teens began HPV vaccination on time (by age 13 years) in 2019, suggesting that more parents are making the decision to protect their teens against HPV-associated cancers. Efforts from federal, state, and other stakeholders to prioritize HPV vaccination among adolescents, and reducing the number of recommended HPV vaccine doses from a 3-dose to a 2-dose series (*2*), likely contributed to these improvements. Coverage with ≥1 dose of MenACWY increased to 88.9%; coverage with ≥2 doses remained low at 53.7%, indicating that continued efforts are needed to improve receipt of the booster dose.

Despite progress in adolescent HPV vaccination and MenACWY coverage, disparities remain; all adolescents are not equally protected against vaccine-preventable diseases. As in previous years, compared with adolescents living in MSA principal cities, HPV UTD status and coverage with ≥1 dose each of HPV vaccine and MenACWY continue to be lower among adolescents in non-MSA areas (*3*). However, these geographic disparities were present only for adolescents at or above the poverty level in 2019. This finding is consistent with another study that found socioeconomic status to be a moderating factor in the association between HPV vaccination and MSA (*4*). The lack of an MSA disparity among adolescents below the poverty level might reflect the access that low-income adolescents have to the VFC program****; previous studies have reported higher HPV vaccination coverage rates among adolescents living below the poverty level (*5*,*6*). Reasons for the MSA disparity among higher socioeconomic status adolescents are less clear but might be an indicator of lower vaccine confidence. More work is needed to understand the relationship between socioeconomic status and geographic disparities and the barriers that might be contributing to such differences.

The findings in this report are subject to at least two limitations. First, the CASRO response rate to NIS-Teen was 19.7%, and only 44.0% of households with completed interviews had adequate provider data. A portion of the questionnaires sent to vaccination provider(s) to request the adolescent’s vaccination history were mailed in early 2020. A lower response rate was observed for those requests, likely because of the effect of the COVID-19 pandemic on health care provider operations.^††††^ Second, even with adjustments for household and provider nonresponse, landline-only households, and phoneless households, a bias in the estimates might remain.^§§§§^

The COVID-19 pandemic has the potential to offset historically high vaccination coverage with Tdap and MenACWY and to reverse gains made in HPV vaccination coverage. Orders for adolescent vaccines have decreased among VFC providers during the pandemic. A recent analysis using VFC provider ordering data showed a decline in vaccine orders for several VFC-funded noninfluenza childhood vaccines since mid-March when COVID-19 was declared a national emergency (*7*). CDC, along with other national health organizations, continues to stress the importance of well-child visits and vaccinations as essential services (*8*). The majority of practices appear to be open and resuming vaccination activities for their pediatric patients (*9*,*10*). Providers can take several steps to ensure that adolescents are up to date with recommended vaccines. These include 1) promoting well-child and vaccination visits; 2) following guidance on safely providing vaccinations during the COVID-19 pandemic^¶¶¶¶^; 3) leveraging reminder and recall systems to remind parents of their teen’s upcoming appointment, and recalling those who missed appointments and vaccinations; and 4) educating eligible patients and parents, especially those who might have lost employer-funded insurance benefits, about the availability of publicly funded vaccines through the VFC program. In addition, state, local, and territorial immunization programs can consider using available immunization information system data***** to identify local areas and sociodemographic groups at risk for undervaccination related to the pandemic, and to help prioritize resources aimed at improving adolescent vaccination coverage.

**Summary**

**What is already known about this topic?**

Three vaccines are routinely recommended for adolescents to prevent diseases that include pertussis, meningococcal disease, and cancers caused by human papillomavirus (HPV).

**What is added by this report?**

Adolescent vaccination coverage in the United States continues to improve for HPV and for meningococcal vaccines, with some disparities. Among adolescents living at or above the poverty level, those living outside a metropolitan statistical area (MSA) had lower coverage with HPV and meningococcal vaccines than did those living in MSA principal cities.

**What are the implications for public health care?**

Ensuring routine immunization services for adolescents, even during the COVID-19 pandemic, is essential to continuing progress in protecting individuals and communities from vaccine-preventable diseases and outbreaks.

## Footnotes

* Eligible participants were born during January 2001–February 2007. Tetanus toxoid, reduced diphtheria toxoid, and acellular pertussis vaccine (Tdap) represents coverage with ≥1 Tdap dose at age ≥10 years. Meningococcal conjugate vaccine (MenACWY) represents coverage with the quadrivalent meningococcal conjugate vaccine or meningococcal-unknown type vaccine. Human papillomavirus (HPV) vaccination coverage includes receipt of any HPV vaccine and does not distinguish between nine-valent (9vHPV), quadrivalent (4vHPV), or bivalent (2vHPV) vaccines. Some adolescents might have received more than the two or three recommended HPV vaccine doses. Estimates for hepatitis A, hepatitis B, and measles, mumps, and rubella vaccines represent coverage based on the catch-up schedule for adolescents who are not up to date with these vaccinations. Except as noted, coverage estimates for ≥1 and ≥2 varicella vaccine doses were obtained among adolescents with no history of varicella disease. Influenza vaccination coverage data are not included in this report but are available online at https://www.cdc.gov/flu/fluvaxview/index.htm.

† Adolescents were considered to be up to date with HPV vaccination if they had received ≥3 doses, or if each of the following applied: 1) they had received 2 doses; 2) the first dose was received before their 15th birthday; and 3) the difference between dates of first and second doses was ≥5 months minus 4 days, the absolute minimum interval between the first and second doses. https://www.cdc.gov/vaccines/programs/iis/cdsi.html.

§ Adolescents were classified as being below the federal poverty level if their total family income was less than the federal poverty level specified for the applicable family size and number of children aged <18 years. All others were classified as at or above the poverty level (https://www.census.gov/data/tables/time-series/demo/income-poverty/historical-poverty-thresholds.html). Poverty status was unknown for 657 adolescents.

¶ Metropolitan statistical area (MSA) status was determined from household reported city and county of residence and was grouped into three categories: MSA principal city, MSA nonprincipal city, and non-MSA. MSA and MSA principal city were as defined by the U.S. Census Bureau (https://www.census.gov/programs-surveys/metro-micro.html). Non-MSA areas include urban populations not located within an MSA and completely rural areas.

** All identified cellular-telephone households were eligible for interview. Sampling weights were adjusted for single frame (cellular telephone), nonresponse, noncoverage, and overlapping samples of mixed telephone users. A description of NIS-Teen single-frame survey methodology and its effect on reported vaccination estimates is available at https://www.cdc.gov/vaccines/imz-managers/coverage/teenvaxview/pubs-presentations/dual-to-single-frame-teen.html.

†† Local areas that received federal immunization funds under Section 317 of the Public Health Service Act were sampled separately. Those included Chicago, Illinois; New York, New York; Philadelphia County, Pennsylvania; Bexar County, Texas; and Houston, Texas. Two local areas were oversampled in 2019: Dallas County, Texas, and El Paso County, Texas. Three territories were sampled separately in 2019: Guam, Puerto Rico, and the U.S. Virgin Islands.

§§ Adolescents from Guam (n = 278), Puerto Rico (n = 216), and U.S. Virgin Island (n = 218) were excluded from the national estimates.

¶¶ The CASRO response rate is the product of three other rates: 1) the resolution rate (the proportion of telephone numbers that can be identified as either for business or residence), 2) the screening rate (the proportion of qualified households that complete the screening process), and 3) the cooperation rate (the proportion of contacted eligible households for which a completed interview is obtained).

*** The NIS-Teen methodology for weighting and synthesizing provider-reported vaccination histories has been previously described. https://www.cdc.gov/vaccines/imz-managers/nis/downloads/NIS-Teen-PUF18-DUG.pdf.

††† Hepatitis A, hepatitis B, varicella, and measles, mumps, and rubella vaccines are considered childhood vaccinations and are recommended for adolescents who are not up to date with these vaccinations. Estimates in this report include those who might have received vaccinations on-time or as catch-up.

§§§ https://www.cdc.gov/vaccines/imz-managers/coverage/teenvaxview/pubs-presentations/NIS-teen-vac-coverage-estimates-2019-tables.html#table-01.

¶¶¶ https://www.cdc.gov/vaccines/imz-managers/coverage/teenvaxview/pubs-presentations/NIS-teen-vac-coverage-estimates-2019-tables.html#table-02.

**** Children aged ≤18 years who are Medicaid-eligible, uninsured, or American Indian/Alaska Native (as defined by the Indian Health Care Improvement Act) are eligible to receive vaccines from providers through the Vaccines for Children (VFC) program. Children categorized as “underinsured” (because their health plans do not include coverage for recommended vaccinations) are eligible to receive VFC vaccines if they are served by a rural health clinic or federally qualified health center or under an approved deputization agreement. https://www.cdc.gov/vaccines/programs/vfc/providers/eligibility.html.

†††† The Provider Record Check (PRC) phase of the NIS, which is conducted in Chicago, was disrupted on March 21, 2020, by a COVID-19–related stay-at-home order issued by the State of Illinois. This disruption meant that some 2019 NIS-Teen data received from responding providers could not be processed and resulted in a lower rate of adolescents with adequate provider data in Quarter 4 among those with consent to contact vaccination providers. NORC at the University of Chicago (https://www.norc.org), the NIS contractor, assessed the effect of the early close of the PRC operation. They found the adequate provider data rate was lower in Quarter 4 than in previous quarters, but that did not affect the demographics of children with adequate provider data or vaccination coverage estimates for MenACWY or HPV vaccines. Logistic regression models indicate that, after controlling for demographic covariates, the odds of being vaccinated with tetanus toxoid, reduced diphtheria toxoid, and acellular pertussis vaccine (Tdap) were lower in Quarter 4 compared with previous quarters, but the effect on the vaccination coverage rate estimate itself was minor.

§§§§ An assessment of validity of the 2018 NIS-Teen estimates has been reported (https://www.cdc.gov/vaccines/imz-managers/nis/downloads/NIS-TEEN-PUF18-DUG.pdf, pages 62–69). NIS-Teen vaccination coverage estimates tended to be slightly low compared with true values derived after adjusting for noncoverage, nonresponse, and vaccination under-acertainment, reaching up to 5.7 percentage points too low for Tdap. This was primarily attributed to under-ascertainment of vaccinations by the NIS provider record check. The validity of estimates did not change from 2017 to 2018.

¶¶¶¶ https://www.cdc.gov/vaccines/pandemic-guidance/index.html.

***** https://repository.immregistries.org/files/resources/5bae51a16a09c/identifying_immunization_pockets_of_need-_final3.pdf.
